# Evaluation of Unified Healthcare Efficiency in China: A Meta-Frontier Non-radial Directional Distance Function Analysis During 2009–2019

**DOI:** 10.3389/fpubh.2022.876449

**Published:** 2022-05-20

**Authors:** Baojie Guo, Jianghua Zhang, Xuemei Fu

**Affiliations:** School of Management, Shandong University, Jinan, China

**Keywords:** healthcare efficiency, non-radial directional distance function, meta-frontier, data envelopment analysis, regional heterogeneity

## Abstract

In this study, we analyze the unified healthcare efficiency in China at the regional level from 2009 to 2019. To accurately evaluate the evolution of unified efficiency from both static and dynamic perspectives, we combine the non-radial directional distance function and the meta-frontier method to evaluate the unified healthcare efficiency and its dynamic changes. This new approach allows for regional heterogeneity and non-radial slack simultaneously. The decomposition of the meta-frontier non-radial Malmquist unified healthcare efficiency index (MNMHEI) can be used to identify the driving factors of dynamic changes. The results show that the unified healthcare efficiency in eastern China is generally higher than that in non-eastern China from the static perspective, implying significant regional differences. Moreover, the unified efficiency in both eastern and non-eastern regions shows similar time trends and reaches the maximum in 2012. From the dynamic perspective, the unified healthcare efficiency increases annually by 2.68% during the study period. This increase in eastern China as a technology leader is mainly driven by technological progress, whereas the increase in non-eastern China is mainly driven by a better catch-up effect. In addition, the impact of the reform on the non-eastern region is more significant for the decreasing technology gap, the stronger growth momentum of technological progress, and global innovative provinces.

## Introduction

After a series of reforms, the healthcare system in China has experienced significant changes. A country's healthcare services are influenced by interrelated political, economic, social, and cultural factors. The continuous changes in these conditions guide the development of healthcare services (1). A well-designed healthcare system can improve the population health conditions, which is conducive to the improvement of national competitiveness. With the economic development and rising overall living standards, China has shown great progress in improving the health status since the reform and opening-up policy in 1978. From 1978 to 2019, life expectancy rises from 66.5 to 77.3 years, and infant mortality drops from 53 to 5.6‰ (2). In the early stages of reform, the strategies compatible with the market economy have brought about the improvement in medical services and also a series of problems, such as the growing inequality among provinces and a rapid increase in healthcare expenditure (3). According to the report on healthcare reform in 2005, the market-oriented healthcare reform was declared unsuccessful for the increasing inequity and decreasing efficiency in health care. The Chinese government started a new round of healthcare reform in 2009, known as the “new healthcare reform.” The fundamental objective of the new reform was to establish a basic medical and healthcare system, supported by four systems, namely, public health, medical service, health care, and drug supply system. To deepen the reform, the government tries to make full use of the advantages of marketization and government intervention. One of the most remarkable achievements of the “new healthcare reform” is that it takes only 3 years for China to achieve universal health insurance coverage. After 2011, the coverage of medical insurance in urban and rural areas has stabilized at above 95%, which increases access to medical services, especially for poor people. Considering problems in the previous unsuccessful reform, it is necessary to examine the evolution of healthcare efficiency, explore regional differences, and identify potential drivers of changes during the post-reform period.

The efficiency evaluation in the healthcare sector after the implementation of the new healthcare reform has attracted considerable attention from scholars. These studies show that although the healthcare efficiency in China has significantly improved since 2009, regional differences always exist (4–6). There is a geographical distribution of healthcare efficiency in China. YU suggests that the regional healthcare efficiency in China is roughly consistent with the level of economic development (7). Economic development may lead to more effective ways of production or management. While China's economy has developed rapidly in the past decade, there remain the challenges, such as unbalanced improvement across different provinces. Thus, the imbalance of economic development may induce significant differences in medical technology levels (8). Despite numerous studies on the evaluation of healthcare efficiency across provinces, we identify a gap in previous research. From a methodological perspective, prior research mainly assesses the healthcare efficiency and explores the regional differences under the assumption of the same production technology, neglecting the technology heterogeneity. Thus, the aim of the study was to evaluate the evolution of unified healthcare efficiency during 2009–2019 through both static and dynamic perspectives based on the technology heterogeneity.

This study has the following contributions. First, we propose an approach combining meta-frontier method and non-radial directional distance function to conduct static and dynamic analyses in unified healthcare efficiency. This approach evaluates the efficiency more accurately by considering the regional technology heterogeneity and the potential non-radial slack simultaneously. Second, we can identify the sources of the changes in unified healthcare efficiency through the decomposition of dynamic indicator (MNMHEI). In addition to the two traditional subcomponents, namely, technological change and efficiency change, we also explore the technology gap change among different regions. Third, we analyze the impact of reforms on unified efficiency changes and explore the emphasis of future policies based on different regions.

The rest of this article is organized as follows. Section Literature Review provides the literature review relevant to our research. Section Methodology introduces the method and data sources. Section Results and Discussion presents the empirical results to analyze both the static and dynamic unified healthcare efficiencies over the period from 2009 to 2019. Section Conclusion concludes the article.

## Literature Review

Healthcare efficiency has been widely studied in different countries using data envelopment analysis (DEA) and Malmquist index from both static and dynamic perspectives. DEA, a nonparametric analysis method developed by Charnes et al. (9), has advantages in evaluating the relative efficiency of decision-making units (DMUs) with multiple outputs and inputs, without assuming a specific functional form. To conduct a temporal analysis of healthcare efficiency, the Malmquist index is generally used to evaluate dynamic changes and identify sources of changes through decomposition, such as “catching up” effect and technological change. Many studies have employed the DEA and Malmquist method to analyze the efficiency in the healthcare sector (10–14).

Recent studies have extended the healthcare efficiency analysis to include the minimization of undesirable outputs, such as mortality and readmissions (15–17). Directional distance function (DDF) is proposed to measure efficiency by maximizing desirable outputs and minimizing undesirable outputs at the same rate. Correspondingly, Chung et al. developed the Malmquist–Luenberger (ML) index to measure the dynamic changes in efficiency evaluated by DDF (18). This ML index has been widely used in measuring environmental performance changes, such as Nakano et al. (19) and Sueyoshi and Goto (20). In terms of healthcare, some scholars have extended the DDF and ML. For example, Falavigna et al. adopted DDF to assess the Italian healthcare efficiency and investigated the influencing factors (21). Gimenez et al. employed the global Malmquist–Luenberger index (GML) to assess the evolution of hospital performance for the post-reform period (22).

However, conventional DDF is considered as a radial model, which may overestimate efficiency when there is slack (23). In addition, it cannot identify the sources of inefficiency for specific indicators (24). In view of the above limitations, non-radial DDF (NDDF) has been developed by allowing for the adjustments of inputs, desirable outputs, and undesirable outputs non-proportionally (25, 26). This novel method has been employed to conduct efficiency analysis in Taiwan's hospitals (15) and Emergency Obstetric of public hospitals in India (27).

With regard to the evolution of efficiency in China's healthcare sector, scholars have explored geographical differences in healthcare efficiency with the development of China's medical reform. Hu suggested that the healthcare efficiency in coastal areas was higher than that in non-coastal areas from 2002 to 2008, but the gap gradually narrowed due to the implementation of the New Rural Cooperative Medical System (NRCMS) in 2003 (28). After the “new healthcare reform” in China, scholars mainly explored the regional differences in healthcare efficiency based on the traditional regional division of the eastern, central, and western areas. By investigating the treatment quality of hospitals in China from 2009 to 2014, Li et al. indicated that the treatment quality in the eastern region was significantly higher than that in the central and western regions, whereas the treatment quality gap between the central region and western region was small (6). Gong et al. assessed the healthcare efficiency from 2009 to 2016 corresponding to the post-reform period, finding a positive correlation between healthcare efficiency and socioeconomic development. He also reported that the eastern region had higher healthcare efficiency than the central region, which in turn had higher healthcare efficiency than the western region (29).

However, a general method in the existing research to explore the regional differences is under the assumption of the same production technology set, neglecting the technology heterogeneity. The neglect of technology heterogeneity may lead to biased results (30). Meta-frontier method is usually proposed to deal with group heterogeneity issue. Therefore, the study examines the healthcare efficiency based on the group heterogeneity to provide comprehensive information.

Through the review of the previous literature, we can find that the healthcare efficiency in China exhibited regional differences. Compared with central and western regions, the efficiency in the eastern region showed better performance due to the advantages in technology, talent, and infrastructure. However, previous studies assessed the healthcare efficiency across different provinces under the assumption of the same production technology set. The neglect of technology heterogeneity may lead to biased results due to China's significant regional gaps. Considering both non-radial slacks and group heterogeneity, this study combines the meta-frontier method and non-radial directional distance function to estimate the unified healthcare efficiency from both the static and dynamic perspectives.

## Methodology

### The Production Technology

Suppose there are *N* decision-making units (DMUs) with information available (in this article, meaning “provinces”), each DMU generates desirable and undesirable outputs through the consumption of inputs. We denote inputs by $x\in R_{+}^{N}$, desirable outputs by $y\in R_{+}^{M}$, and undesirable outputs by $b\in R_{+}^{I}$. In general, we can define the production technology set as follows:


(1) $$\begin{array}{l} T=\left\{\left(x,y,b\right):\;x\;can\;produce\;\left(y,b\right)\right\} \end{array}$$

One can refer to Fare and Grosskopf for the standard axioms of production theory (31). In this study, referring to the method of Zhou et al. (26), we can formulate the production sets T with constant returns to scale in the following way.


(2) $$\begin{array}{l} T=\left\{\begin{array}{l} \left(x,y,b\right):\overset{N}{\underset{n=1}{\sum }}z_{n}x_{n}\leq x,\overset{N}{\underset{n=1}{\sum }}z_{n}y_{n}\geq y,\;\\ \;\overset{N}{\underset{n=1}{\sum }}z_{n}b_{n}=b,z_{n}\geq 0,n=1,2,\ldots \ldots ,N \end{array}\right\} \end{array}$$

where the row vector *Z*_*n*_ is an intensity variable, which constructs a set of production technologies using a convex combination. Based on Tulkens and Vanden Eeckaut (32) and Oh and Lee (33), we distinguish three production technology sets according to the concepts of meta-frontier and group frontier: contemporary production technology, intertemporal production technology, and global production technology.

Suppose there are *H* groups showing technological heterogeneity, the contemporary production technology indicates the specific technology of the group frontier at a specific time. For the group *h* at a specific period *t*, the contemporary production technology can be defined as follows:


(3) $$\begin{array}{l} T_{Rh}^{C}=\left\{\begin{array}{l} \left(x^{t},y^{t},b^{t}\right):\\ \left(x^{t}\right)\;can\;produce\;(y^{t},b^{t})\;where\;t=1,\ldots T. \end{array}\right\} \end{array}$$

The intertemporal production technology consists of all the observations that cover the whole period for the specific group *h*. It indicates the specific technology of the group frontier over the whole period. We define the above technology as follows:


(4) $$\begin{array}{l} T_{Rh}^{I}=T_{Rh}^{1}\cup T_{Rh}^{2}\cup \ldots T_{Rh}^{T} \end{array}$$

The global production technology contains observations from all groups during all sample periods, representing the technology of meta-frontier. It is the aggregation of all intertemporal production technology sets and can be defined as follows:


(5) $$\begin{array}{l} T^{G}=T_{R1}^{I}\cup T_{R2}^{I}\cup \ldots T_{Rh}^{I} \end{array}$$

### Variables

The purpose of an efficient healthcare system was to provide more medical services and improve the residents' health status with limited resources. Death is inevitable in the process of providing services.

In addition, the improvement in health level manifests in the improvement in maternal and child hygiene levels, and disease control levels (34). Thus, it is important to consider deaths as undesirable outputs.

According to the literature review of healthcare efficiency from the study of Kohl et al. (35) and Ozcan (36), previous studies mainly select labor, capital investment, operating expenses as input variables, and outpatient visits, inpatient visits as output variables. The capital investment can generally be proxied by beds in previous studies. However, some scholars have criticized the mixed-use of economic indicators and quantitative indicators due to the confusion between technical efficiency and allocative efficiency (9, 37). Thus, the economic indicators have not been adopted into the input–output indicators. Based on the importance and availability of indicators, the inputs, desirable outputs, and undesirable outputs are specified as follows. The inputs *x* selected in our study include the number of health technicians (E) and the number of beds (B). Healthcare output is complicated, which reflects the level of health service delivery, disease control, maternal and perinatal hygiene, and so on. Accordingly, the number of outpatient visits (O) and the number of inpatient visits (I) are measured as desirable outputs *y*. Undesirable outputs *b* selected are three types of death to reflect the competitiveness of each province in the health care: maternal mortality (M), perinatal mortality (P), and contagious mortality (C).

### Non-radial Directional Distance Function

This article applies the non-radial directional distance function (NDDF) to the healthcare efficiency evaluation in China (26, 38). The description of the NDDF in the case of healthcare sector can be specified as follows:


(6) $$\begin{array}{l} \vec{D}\left(x,y,b;g\right)=\sup\left\{w^{T}\beta \;:\left(\left(x,y,b\right)+g\cdot diag\left(\beta \right)\right)\in T\right\} \end{array}$$

Here, $w^{T}={\left(w_{x},w_{y},w_{b}\right)}^{T}$ is the vector of exogenous weights assigned to inputs and outputs; *g* = (−*g*_*x*_, *g*_*y*_, −*g*_*b*_) denotes the directional vector in which the inputs, desirable outputs, and undesirable outputs will be scaled. $\beta ={\left(\beta_{x},\beta_{y},\beta_{b}\right)}^{T}\geq 0$ represents the vector of scaling factors. The calculation of NDDF value for a specific province *n*′ can be obtained by solving the following linear program.


(7) $$\begin{array}{l} \vec{D}\left(x,y,b;g\right)=\max w_{x}\beta_{x}+w_{y}\beta_{y}+w_{b}\beta_{b}\\ \;s.t.\overset{N}{\underset{n=1}{\sum }}z_{n}x_{n}\leq x_{n^{\prime }}-\beta_{x}g_{x}\;\\ \;\overset{N}{\underset{n=1}{\sum }}z_{n}y_{n}\geq y_{n^{\prime }}+\beta_{y}g_{y}\;\\ \;\overset{N}{\underset{n=1}{\sum }}z_{n}b_{n}=b_{n^{\prime }}-\beta_{b}g_{b}\\ \;z_{n}\geq 0,\beta_{x},\beta_{y},\beta_{b}\geq 0 \end{array}$$

If $\vec{D}\left(x,y,b;g\right)$=0, it means that the inefficiency level of this DMU is zero in the *g* direction and the DMU is located on the best-practice frontier.

Because there are two inputs (health technicians, beds), two desirable outputs (outpatient visits, inpatient visits), and three undesirable outputs (maternal mortality, perinatal mortality, contagious mortality), we set the directional vector *g* = (−*E*, −*B, O, I*, −*M*, −*P*, −*C*) and the weight vector *w*^*T*^ equal to$\left(\frac{1}{6},\frac{1}{6},\frac{1}{6},\frac{1}{6},\frac{1}{9},\frac{1}{9},\frac{1}{9}\right)$. This study employs the method introduced by Zhou et al. (26) to construct a static unified efficiency index. We define a unified healthcare efficiency index (HEI) to carry out static analysis in China. If $\beta_{E}^{*},\beta_{B}^{*},\beta_{O}^{*},\beta_{I}^{\text{*}},\beta_{M}^{*},\beta_{P}^{*},\beta_{C}^{*}$ are optimal solutions in the model (7), then HEI can be expressed as Equation (8). The HEI ranges from zero to unity. The higher the HEI value, the higher the unified healthcare efficiency is. If the HEI equals to unity, it means that the DMU is located on the best-practice frontier.


(8) $$\begin{array}{l} \text{HEI=}\frac{1-\frac{1}{5}\left(\beta_{E}^{*}+\beta_{B}^{*}+\beta_{M}^{*}+\beta_{P}^{*}+\beta_{C}^{*}\right)}{1+\frac{1}{2}\left(\beta_{O}^{*}+\beta_{I}^{*}\right)} \end{array}$$

### Meta-Frontier Non-radial Malmquist Unified Healthcare Efficiency Index

In the Section The Production Technology, we described three production technology sets according to the concepts of meta-frontier and group frontier. Then, we incorporate the three production technology sets into the NDDF model (7) and obtain corresponding NDDF models. Suppose there are *H* groups, then, the contemporary NDDF model for a specific group *R*_*h*_ at a specific time *t* is given by:


(9) $$\begin{array}{l} {\vec{D}}^{C}\left(x,y,b;g\right)=\sup\left\{w^{T}\beta^{C}\;:\left(\left(x,y,b\right)+g\cdot diag\left(\beta^{C}\right)\right)\in T_{Rh}^{C}\right\} \end{array}$$

The intertemporal NDDF for a specific group *R*_*h*_ covering the whole periods is given by:


(10) $$\begin{array}{l} {\vec{D}}^{I}\left(x,y,b;g\right)=\sup\left\{w^{T}\beta^{I}\;:\left(\left(x,y,b\right)+g\cdot diag\left(\beta^{I}\right)\right)\in T_{Rh}^{I}\right\} \end{array}$$

Analogously, the global NDDF covering all groups and sample periods is given by:


(11) $$\begin{array}{l} {\vec{D}}^{G}\left(x,y,b;g\right)=\sup\left\{w^{T}\beta^{\text{G}}\;:\left(\left(x,y,b\right)+g\cdot diag\left(\beta^{G}\right)\right)\in T^{G}\right\} \end{array}$$

We define the meta-frontier non-radial Malmquist unified healthcare efficiency index (MNMHEI) to conduct the dynamic analysis by incorporating the NDDF model into the meta-frontier Malmquist index. To evaluate the dynamic changes in the unified efficiency and decompose the MNMHEI, we need to solve NDDF models under three different production technology sets ${\vec{D}}^{C}\left(x,y,b\right)$, ${\vec{D}}^{I}\left(x,y,b\right)$, ${\vec{D}}^{G}\left(x,y,b\right)$ during two adjacent periods, i.e., *t* and *t* + 1. The calculation of ${\vec{D}}^{d}\left(x^{t},y^{t},b^{t}\right)$ corresponding to three types of technology sets at a specific time *t* is made by solving the following linear program.


(12) $$\begin{array}{l} {\vec{D}}^{d}\left(x^{t},y^{t},b^{t};g\right)=\max w_{x}\beta_{x}^{d,t}+w_{y}\beta_{y}^{d,t}+w_{b}\beta_{b}^{d,t}\\ \;s.t.\underset{con}{\sum }z_{n}^{d,t}x_{n}^{d,t}\leq x_{n^{\prime }}^{t}-\beta_{x}^{d,t}g_{x}^{t}\;\\ \;\underset{con}{\sum }z_{n}^{d,t}y_{n}^{d,t}\geq y_{n^{\prime }}^{t}+\beta_{y}^{d,t}g_{y}^{t}\\ \;\underset{con}{\sum }z_{n}^{d,t}b_{n}^{d,t}=b_{n^{\prime }}^{t}-\beta_{b}^{d,t}g_{b}^{t}\;\\ \;z_{n}^{d,t}\geq 0,\beta_{x}^{d,t},\beta_{y}^{d,t},\beta_{b}^{d,t}\geq 0 \end{array}$$

The superscript *d* indicates three types of NDDF models mentioned above. Here, *d* = (*C, I, G*). The symbol *con* under ∑ represents conditions corresponding to three different production technology sets. If *d* = *C*, we can obtain the contemporary NDDF and $con=T_{Rh}^{C}$. If *d* = *I*, we can obtain the intertemporal NDDF and $con=T_{Rh}^{I}$. If *d* = *G*, the global NDDF is defined with $con=T^{G}$. Based on Equations (8) and (12), the HEI^d^ of a DMU corresponding to three different NDDFs at period *t* can be generated, denoted as HEI^C^(*t*), HEI^I^(*t*), and HEI^G^(*t*).


(13) $$\begin{array}{l} HEI^{d}\left(x^{t},y^{t},b^{t}\right)=\frac{1-\frac{1}{5}\left(\beta_{E}^{d*,t}+\beta_{B}^{d*,t}+\beta_{M}^{d*,t}+\beta_{P}^{d*,t}+\beta_{C}^{d*,t}\right)}{1+\frac{1}{2}\left(\beta_{O}^{d*,t}+\beta_{I}^{d*,t}\right)}\\ d=\left(C,I,G\right) \end{array}$$

Analogously, we can also generate three kinds of HEI at period *t*+1 by replacing *t* with *t*+1 in Equation (13), denoted as HEI^C^(*t*+1), HEI^I^(*t*+1), and HEI^G^(*t*+1), respectively. Then, we define the meta-frontier non-radial Malmquist index for unified healthcare efficiency (MNMHEI) based on the global production technology set (*T*^*G*^) in the following way. It is used to measure the dynamic changes of the unified healthcare efficiency from time *t* to *t*+1.


(14) $$\begin{array}{l} MNMHEI\left(t,t+1\right)\text{=}\frac{HEI^{G}\left(x^{t+1},y^{t+1},b^{t+1}\right)}{HEI^{G}\left(x^{t},y^{t},b^{t}\right)} \end{array}$$

If MNMHEI is greater than unity, the DMU in *t*+1 is closer to the global frontier than that in *t*, which represents an improvement in unified efficiency. If MNMHEI is less than unity, the DMU in *t*+1 is farther away from the global frontier than that in *t*, implying a deterioration during the corresponding period. Referring to Oh and Lee (33), Equation (14) can be decomposed into three parts to identify potential drivers as follows:


(15) $$\begin{array}{l} MNMHEI\left(t,t+1\right)=\frac{HEI^{G}\left(\cdot^{t+1}\right)}{HEI^{G}\left(\cdot^{t}\right)}\\ =\left[\frac{HEI^{C}\left(\cdot^{t+1}\right)}{HEI^{C}\left(\cdot^{t}\right)}\right]*\left[\frac{HEI^{I}\left(\cdot^{t+1}\right)/HEI^{C}\left(\cdot^{t+1}\right)}{HEI^{I}\left(\cdot^{t}\right)/HEI^{C}\left(\cdot^{t}\right)}\right]*\left[\frac{HEI^{G}\left(\cdot^{t+1}\right)/HEI^{I}\left(\cdot^{t+1}\right)}{HEI^{G}\left(\cdot^{t}\right)/HEI^{I}\left(\cdot^{t}\right)}\right]\\ =\left[\frac{TE^{t+1}}{TE^{t}}\right]*\left[\frac{BPR^{t+1}}{BPR^{t}}\right]*\left[\frac{TGR^{t+1}}{TGR^{t}}\right]\\ =EC^{t,t+1}*BPC^{t,t+1}*TGC^{t,t+1} \end{array}$$

where EC^*t,t*+1^ (efficiency change) captures the technical efficiency changes relative to contemporary frontier from time *t* to *t*+1. EC^*t,t*+1^ >1 represents an improvement in technical efficiency, since the inefficiency distance from a DMU to the contemporary frontier in *t*+1 is smaller than that in t. If EC^*t,t*+1^ <1, it indicates an efficiency decrease. BPC^*t,t*+1^( the best-practice gap change) measures frontier shifts between contemporary technology frontier and intertemporal technology frontier from *t* to *t*+1. BPC^*t,t*+1^ >1 indicates that the contemporary frontier moves toward the intertemporal frontier, representing technological progress and innovation effect. BPC^*t,t*+1^ <1 represents that the contemporary frontier shifts far away from the intertemporal frontier, implying technological deterioration.

TGC^*t,t*+1^ (technology gap change) measures changes in technology gap between intertemporal frontier and global frontier during two periods. This indicator is the ratio of TGR in year *t*+1 to that in year *t*. If TGR =1, the HEI calculated by the intertemporal frontier NDDF is equal to that calculated by the global frontier NDDF, implying the leading position in inventing new technologies. The group which has more DMUs with TGR = 1 is the leading group (33). Correspondingly, TGC^*t,t*+1^ collects the changes in technology leadership. If TGC^*t,t*+1^ >1, it represents a decrease in the technology gap between the aforementioned two frontiers. TGC^*t,t*+1^ <1 is interpreted conversely.

### Data

We adopt the method mentioned above to evaluate the unified healthcare efficiency in China from 2009 to 2019. The sample consists of 31 provinces which can be divided into two large groups (eastern region and non-eastern region) based on geographical and economic characteristics. The eastern region includes eleven relatively developed coastal provinces: Beijing, Tianjin, Hebei, Liaoning, Shanghai, Jiangsu, Zhejiang, Fujian, Shandong, Guangdong, and Hainan. The remaining 20 provinces belong to the relatively underdeveloped non-eastern region. The data for this study are drawn from China Health Statistic Yearbook.

Table 1 presents the statistical description results of the input and output variables. For the input variables and desirable output variables, the average values in eastern region are markedly higher than those in non-eastern region. Both groups show significant growth during the sample period. Except outpatient visits (O), other inputs and desirable outputs in non-eastern region have higher annual growth rates than those in eastern region. In addition, we should pay more attention to the undesirable output variables because of the noticeable differences between the two groups. For all the undesirable outputs, the average values in non-eastern region are significantly higher than those in the eastern region. Annual growth rates of maternal mortality (M) and perinatal mortality (P) in two groups are both negative, representing the improvement in health level. In comparison with the eastern region, non-eastern China experiences a more obvious decline. Contagious mortality (C) shows the greatest differences in annual growth rate. Average annual growth rate in eastern region is −1.86%, while that in non-eastern region is 6.2%. It can be found that the contagious mortality in the non-eastern region has a clear upward trend, which is different from the slight downward trend in eastern region.

**Table 1:** Descriptive statistics of input and output data (2009–2019).

**Type**	**Variables**	**Unit**	**Groups**	**Mean**	**SD**	**Min**	**Max**	**Annual growth rate**
Inputs	E	Thousand people	ES	307.10	193.16	37.86	792.59	6.19%
			NE	213.49	132.20	9.34	653.89	6.28%
	B	Thousand	ES	237.50	157.73	23.53	629.72	6.34%
			NE	198.68	131.81	8.35	640.15	7.69%
Desirable outputs	O	10 thousand people	ES	33636.97	22290.68	3127.32	89179.77	5.08%
			NE	17970.96	13215.42	959.19	61020.29	4.40%
	I	10 thousand people	ES	693.75	497.54	63.54	1849.93	6.84%
			NE	617.54	438.74	14.40	2013.22	7.30%
Undesirable outputs	M	Per 100 thousand people	ES	9.07	4.58	1.1	25.8	−5.03%
			NE	24.73	28.75	6.4	232.2	−8.42%
	P	‰	ES	4.94	1.73	1.8	9.61	−5.79%
			NE	7.22	3.84	2.28	24.04	−6.59%
	C	Per 100 thousand people	ES	0.64	0.31	0.22	1.69	−1.86%
			NE	1.62	1.64	0.26	8.2	6.2%

*ES, eastern; NE, non-eastern*.

## Results and Discussion

### Analysis of the Static HEI^d^

Healthcare efficiency index is generated to evaluate the static unified healthcare efficiency. As explained above, we consider three categories of HEI^d^: HEI^G^, HEI^I^, and HEI^C^, which are measured under global frontier, intertemporal frontier, and contemporary frontier, respectively. Since the three different production technology sets satisfy the following relationship: $T_{Rh}^{C}\subseteq T_{Rh}^{I}\subseteq T^{G}$, the inefficiency distance between a DMU and global frontier is greater than or equal to that between a DMU and intertemporal frontier. Analogously, the inefficiency distance between a DMU and intertemporal frontier is not less than that between a DMU and contemporary frontier. Then, HEI^G^ ≤ HEI^I^ ≤ HEI^C^ always holds.

**Figure 1:**
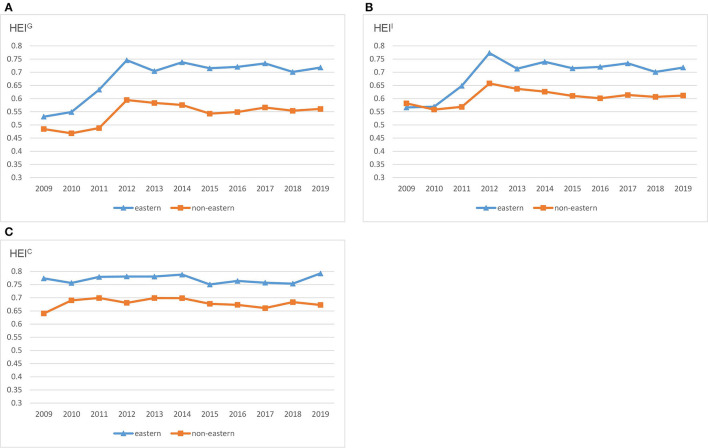
HEI^d^ across regions in China. **(A)** HEI^G^. **(B)** HEI^I^. **(C)** HEI^C^.

Figure 1 provides the empirical results of three average HEI in two regions from 2009 to 2019. In Figure 1A, HEI^G^ values in the eastern region are obviously higher than those in non-eastern region during the whole study period, implying significant regional differences. It is interesting to find that HEI^G^ values in eastern region show similar time trend to that in non-eastern region. HEI^G^ in two regions exhibits the largest increase from 2011 to 2012 and reaches the maximum in 2012 simultaneously. The most likely reason is the whole coverage of basic health insurance of China in 2011 (39), which improves the access to health services for people with health insurance. After 2012, the HEI^G^ values remain stable with the trend of fluctuation.

These results are consistent with Gong et al. (29) who indicate that the healthcare efficiency in 2012 is the highest during 2009–2016 due to the universal health insurance coverage. With respect to the regional differences, they also show that the healthcare efficiency in the eastern region is the highest among the three regions in China (29). Even though the time trend of healthcare efficiency in the two regions is similar, the gap always exists and remains stable, which is different from the study on the gradual narrowing of regional gap during 2002–2008.

As shown in Figure 1B, the spatial and temporal distribution patterns of HEI^I^ are similar to those of HEI^G^. Figure 1C describes the time trend of HEI^C^ under contemporary frontier. HEI^C^ in the two regions keeps stable with less fluctuation during 2009–2019. Moreover, HEI^C^ values in eastern region are higher than those in non-eastern region. Overall, the two regions show similar time trends of HEI under the influence of policies.

### Analysis of MNMHEI and the Decomposition

#### MNMHEI and the Decomposition at the Regional and Provincial Level

The dynamic changes in unified healthcare efficiency are evaluated by MNMHEI. Table 2 reports the results of average MNMHEI and its decomposition for each of the provinces. The average MNMHEI in China is 1.0268, implying that the unified healthcare efficiency under the global frontier increases annually by 2.68% from 2009 to 2019. Both eastern and non-eastern regions show an upward trend, with an average growth rate of 3.53 and 2.22%, respectively. At the provincial level, one province (9.1%) in eastern China and four provinces (20%) in non-eastern China exhibit a decline. Among all provinces, Hubei shows the largest increase (8.67%) whereas Guizhou is the province that demonstrates the largest decrease (−4.26%).

**Table 2:** Average MNMHEI and the decomposition of provinces in China.

**Province**	**Area**	**MNMHEI**	**EC**	**BPC**	**TGC**
Beijing	ES	1.0704	1.1299	0.9776	1.0000
Tianjin	ES	1.0318	1.0163	1.0159	1.0000
Hebei	ES	1.0411	1.0033	1.0419	1.0000
Liaoning	ES	1.0113	0.9839	1.0300	1.0000
Shanghai	ES	1.0758	1.0000	1.0758	1.0000
Jiangsu	ES	1.0438	1.0000	1.0438	1.0000
Zhejiang	ES	1.0646	1.0000	1.0646	1.0000
Fujian	ES	0.9912	0.9607	1.0210	1.0548
Shandong	ES	1.0453	1.0000	1.0453	1.0000
Guangdong	ES	1.0050	1.0000	1.0050	1.0000
Hainan	ES	1.0077	1.0022	1.0060	1.0000
Shanxi	NE	1.0128	1.0001	1.0248	0.9915
Inner Mongolia	NE	1.0132	1.0161	1.0089	0.9982
Jilin	NE	1.0109	1.0049	1.0099	0.9967
Heilongjiang	NE	1.0105	0.9830	1.0232	1.0061
Anhui	NE	1.0434	1.0345	1.0339	1.0035
Jiangxi	NE	0.9930	1.0000	0.9906	1.0004
Henan	NE	1.0679	1.0000	1.0389	1.0298
Hubei	NE	1.0867	1.0249	1.0360	1.0270
Hunan	NE	1.0437	1.0113	1.0219	1.0129
Guangxi	NE	1.0718	1.0000	1.0171	1.0485
Chongqing	NE	1.0186	1.0959	0.9786	1.0062
Sichuan	NE	1.0092	1.0000	1.0072	1.0002
Guizhou	NE	0.9574	0.9437	1.0434	1.0001
Yunnan	NE	1.0372	0.9551	1.0338	1.0709
Xizang	NE	0.9951	1.0163	0.9857	0.9978
Shaanxi	NE	1.0178	1.0160	1.0347	0.9977
Gansu	NE	1.0299	1.0205	1.0059	1.0087
Qinghai	NE	0.9843	0.9981	0.9962	0.9904
Ningxia	NE	1.0058	1.1665	0.9411	0.9927
Xinjiang	NE	1.0347	1.0185	0.9981	1.0180
Eastern region		1.0353	1.0088	1.0297	1.0050
Non-eastern region		1.0222	1.0153	1.0115	1.0099
China		1.0268	1.0130	1.0180	1.0081

*ES, eastern; NE, non-eastern*.

To reveal the sources of the unified healthcare efficiency changes, the MNMHEI is decomposed into efficiency change (EC), best-practice gap change (BPC), and technology gap change (TGC). EC reveals how a DMU's proximity to the contemporary frontier changes during the sample period, implying the catch-up effect. The average EC has a value of 1.013, indicating an average annual growth of 1.3%. This means that the provinces generally move toward the contemporary frontier from 2009 to 2019. At the regional level, non-eastern China shows more significant growth than does eastern China, with the average growth rate of 1.53 and 0.88%, respectively. At the provincial level, the province with the best catch-up effect is Ningxia (16.65%), whereas Guizhou shows the poorest catch-up effect (−5.63%). A number of two provinces (18%) in eastern China and three provinces (20%) in non-eastern China experience a decline in EC.

Best-practice gap change measures frontier shifts, implying technological change during the period. The average BPC is 1.018, larger than unity, which means that technological progress has taken place on average. Contrary to EC at the regional level, the average BPC value in eastern region (1.0297) is higher than that in non-eastern region (1.0115). It indicates that eastern China shows better performance in innovation compared with the non-eastern region. For individual provinces, a total of 24 provinces experience technological progress, whereas only 7 provinces show a technological decline. The BPC in Shanghai has the highest value (1.0758), whereas the BPC in Ningxia has the lowest value (0.9411). For most provinces in eastern China, the improvement in unified healthcare efficiency is accompanied by technological progress. However, provinces that experience technological deterioration are mainly located in the non-eastern region.

**Figure 2:**
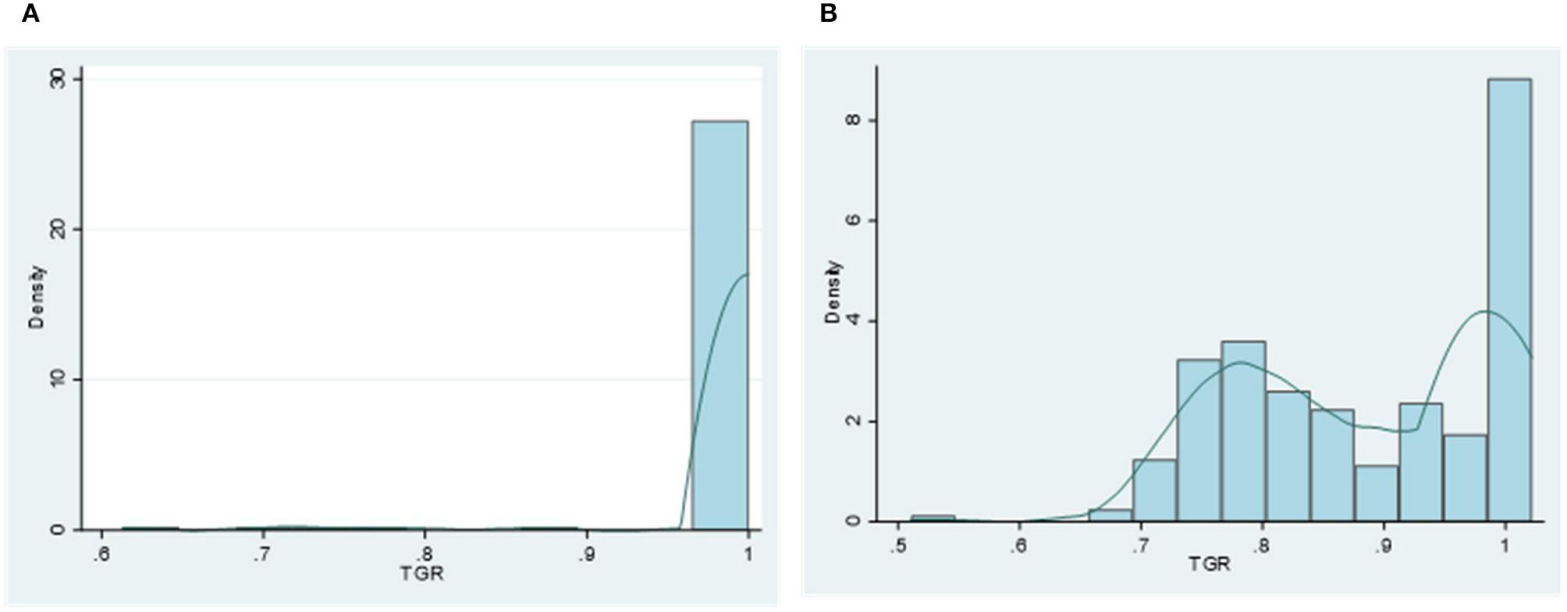
Histogram and kernel density estimation of each region's technical gap ratio (TGR). **(A)** Eastern region. **(B)** Non-eastern region.

The average TGC in China is 1.0081, implying less change in the gap between the intertemporal frontier and the global frontier. An interesting result is that the average TGC values in most eastern provinces are equal to unity, which means that the technology gaps in those provinces between intertemporal frontier and global frontier keep stable. However, the information provided by TGC only includes the dynamic changes in the technology gap, but not the static values of technology gap. To solve this argument, we plot the histogram of TGR for each region, reflecting the distribution of static technology gap. If TGR equals to unity, there is no gap between intertemporal frontier and global frontier for the specific group. The smaller the value of TGR, the larger the gap between intertemporal frontier and global frontier. According to the distribution of TGR in Figure 2A, the TGR values in most eastern provinces are equal to unity, indicating that eastern China is the leading group. In Figure 2B, the distribution of TGR in non-eastern provinces is more dispersed than that in eastern provinces. In addition to a large number of data aggregations around 1, there is also some aggregations around 0.8. This evidence means that the gaps between intertemporal frontier and global frontier in non-eastern provinces are larger than those in the eastern provinces. Even though non-eastern China is considered as the technology follower, the average TGC values of most provinces (65%) in this region are greater than unity, indicating a decreasing technology gap between this group intertemporal frontier and global frontier during the sample period (as shown in Table 2).

#### Temporal Analysis of MNMHEI and the Decomposition

**Figure 3:**
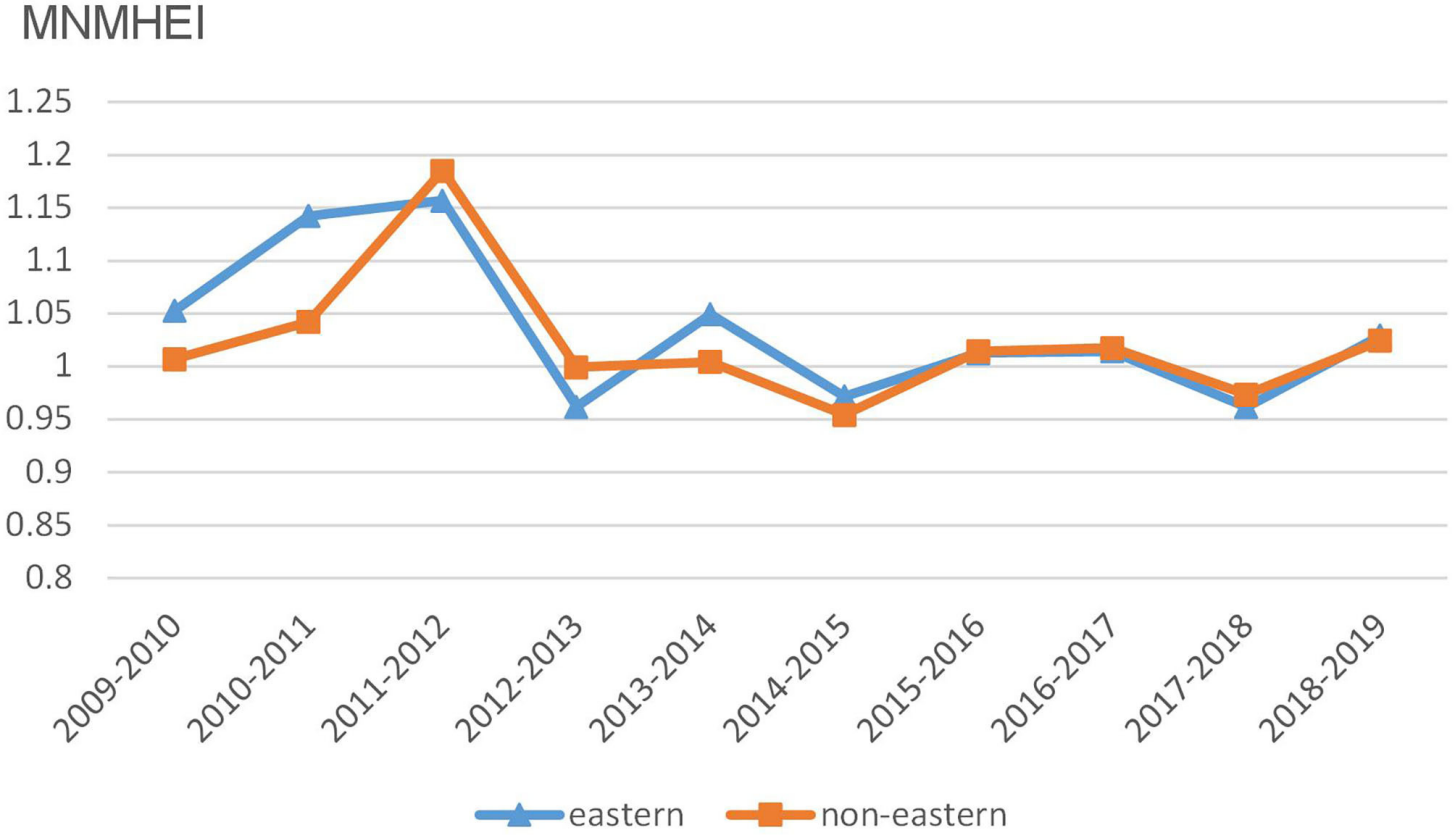
Trends of MNMHEI at the regional level.

We also examine time trends of dynamic changes in the unified healthcare efficiency and its decomposition under the MNMHEI framework. Figure 3 reports time trends of MNMHEI at the regional level. Both eastern and non-eastern China share similar time trends of MNMHEI during the sample period. Between 2011 and 2012, the MNMHEI in two regions shows the greatest values. As mentioned above, the notable growth in unified healthcare efficiency is a combined result of universal health insurance coverage and a low comparison base. In the following years, the MNMHEI in two regions experiences a significant decline from 2012 to 2013 with values less than unity, followed by a slight fluctuating trend.

**Figure 4:**
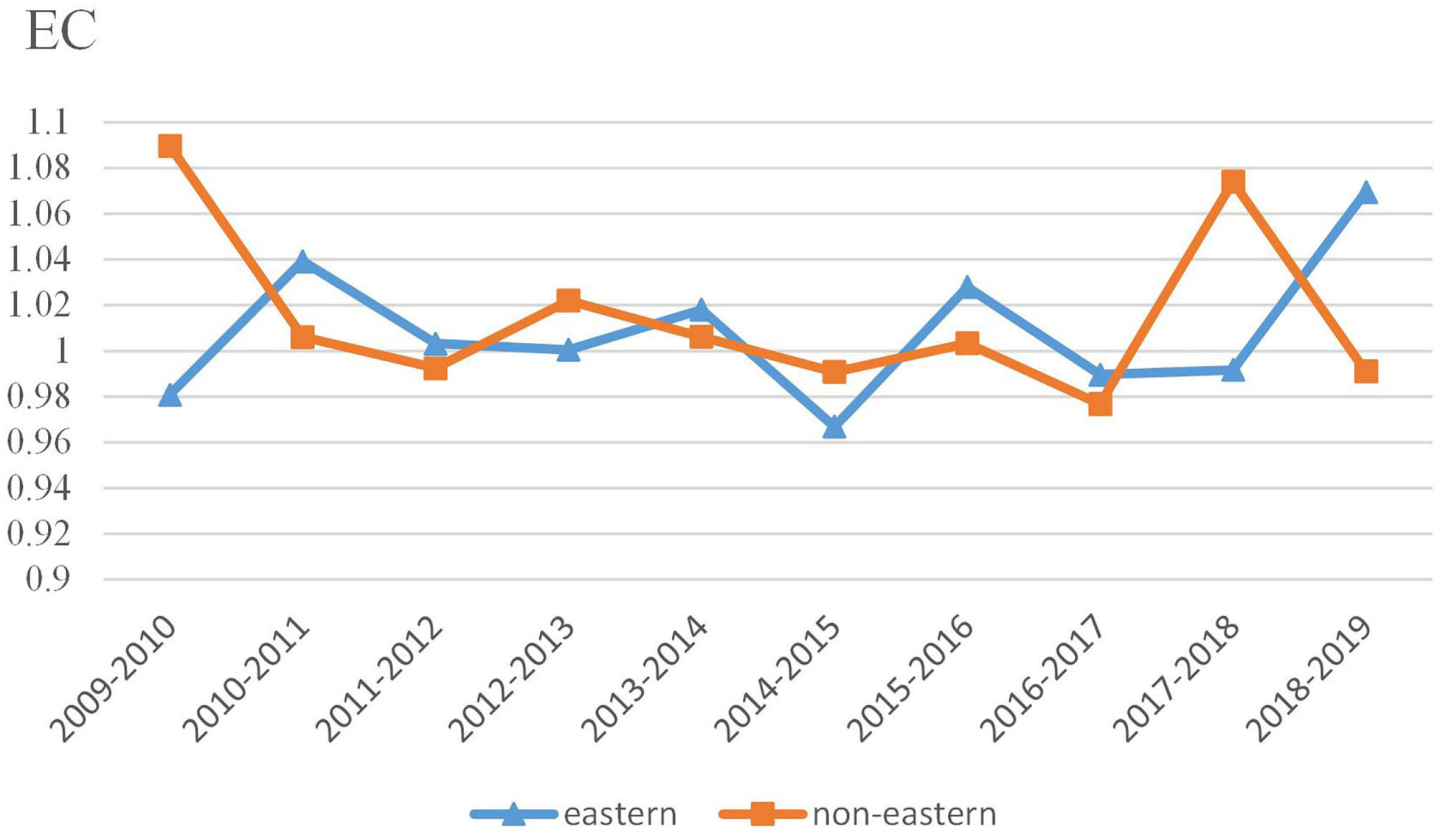
Trends of EC at the regional level.

Figure 4 reports average EC values in eastern and non-eastern regions from 2009 to 2019. Time trends of EC values in two regions fluctuate both up and down, without obvious upward or downward trends. It is found that there is a slightly competitive relationship of EC values between eastern China and non-eastern China since the ranking changes each period. For example, the eastern group shows higher values in six periods whereas the non-eastern group performs better in four periods.

**Figure 5:**
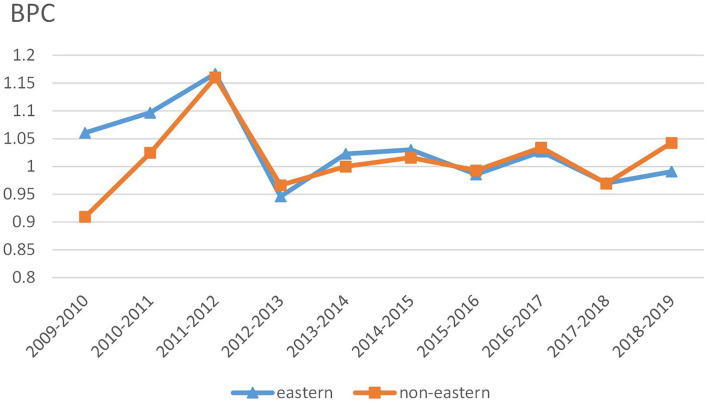
Trends of BPC at the regional level.

In Figure 5, the BPC values in eastern China show a similar time trend with those in non-eastern China. In addition, the BPC time trends in two regions approximately coincide with the changes in MNMHEI according to the results in Figures 3, 5. Both MNMHEI values and BPC values show a marked increase from 2011 to 2012. The phenomenon may emerge from China's policy of universal health insurance coverage which has a positive impact on technological progress. The eastern region shows higher BPC values from 2009 to 2012, whereas there is no significant difference in BPC between eastern and non-eastern China from 2012 to 2018. After 2018, the BPC in the non-eastern region performs better. It is interesting to note that even though the average BPC value in eastern region is higher during the whole study period (as shown in Table 2), the BPC in non-eastern region shows a stronger growth momentum from the perspective of time trend.

**Figure 6:**
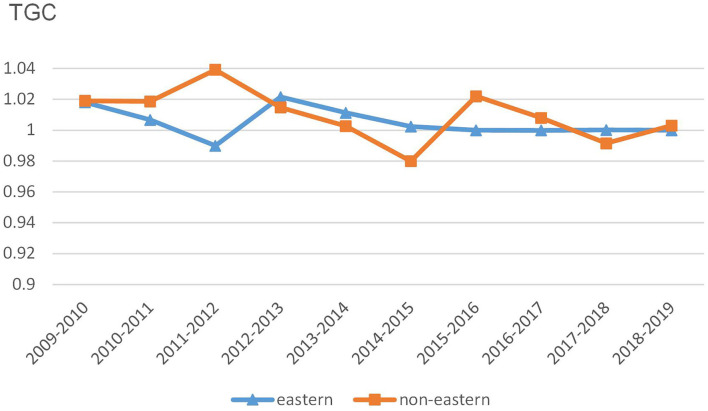
Trends of TGC at the regional level.

As mentioned above, TGC measures the changes in technology gap for the specific group. Figure 6 describes TGC values of eastern and non-eastern China during 2009–2019. On the whole, TGC values in the non-eastern region show a greater fluctuation trend than those in the eastern region. From 2009 to 2013, we observe almost opposite time trends between eastern and non-eastern regions. Non-eastern region during 2011–2012 shows the greatest value of TGC, implying a significant decreasing technology gap between the intertemporal frontier and the global frontier. However, the eastern region experiences a widening technology gap in the same time frame. It seems that the policy of universal health insurance coverage achieved in 2011 has a more positive effect on reducing the technology gap in the relatively underdeveloped non-eastern region. After 2014, the TGC values in the eastern region remain relatively stable, whereas there is still a large fluctuation trend of TGC in the non-eastern region. Although the TGC values in the eastern region fluctuate slightly in the early stage of the sample period, the eastern region has maintained the leader of technology due to the existence of a large number of TGR values equal to 1. For the non-eastern region, the TGC values are larger than unity in most cases regardless of the obvious fluctuation, implying the decreasing technology gap during the whole study period.

### Innovative Provinces

Referring to Oh and Lee (33) and Zhang and Choi (40), we identify two types of innovative provinces that can be targeted by those inefficient provinces: the regional innovative provinces and the global innovative provinces. Regional innovative provinces can be regarded as innovators within a specific group, and global innovative provinces include a subset of regional innovators from an integrated perspective. A total of three conditions for identifying regional innovative provinces are described as follows:


(16a) $$\begin{array}{l} BPC>1 \end{array}$$

(16b) $$\begin{array}{l} {\vec{D}}^{\text{t}}\left(T^{t+1},B^{t+1},O^{t+1},I^{t+1},M^{t+1},P^{t+1},C^{t+1}\right)<0 \end{array}$$

(16c) $$\begin{array}{l} {\vec{D}}^{\text{t+1}}\left(T^{t+1},B^{t+1},O^{t+1},I^{t+1},M^{t+1},P^{t+1},C^{t+1}\right)\text{=}0 \end{array}$$

As described earlier, Equation (16a) indicates that the contemporary frontier in *t*+1 shifts closer to the intertemporal frontier than that in *t*, representing an innovation effect. Equation (16b) suggests that the production of innovative provinces in period *t*+1 should be outside of the contemporary production possibility set of period *t*. It also means that the technology in period *t* cannot satisfy the required production activity in period *t*+1. Equation (16c) indicates that the regional innovative provinces in period *t*+1 should be completely efficient under the contemporary frontier in period *t*+1.

In addition to the three conditions mentioned above, we add two additional conditions to identify the global innovative provinces.


(17a) $$\begin{array}{l} TGC>1 \end{array}$$

(17b) $$\begin{array}{l} {\vec{D}}^{G}\left(T^{t+1},B^{t+1},O^{t+1},I^{t+1},M^{t+1},P^{t+1},C^{t+1}\right)\text{=}0 \end{array}$$

Equation (17a) indicates a decrease in the technology gap between the intertemporal frontier and the global frontier, implying technology convergence toward the global frontier. Equation (17b) suggests that the global innovative provinces in period t+1 should be completely efficient under the global frontier.

Table 3 lists the regional innovative provinces and global innovative provinces. In eastern China, most of the regional innovation provinces are mainly concentrated in the Yangtze River Delta region, such as Shanghai, Jiangsu, and Zhejiang. Among them, Zhejiang appears seven times as the province with the highest frequency. Shanghai and Jiangsu appear five and four times, respectively. In addition, Shandong and Guangdong are also identified as regional innovative provinces four and three times, respectively. In non-eastern China, regional innovative provinces vary across periods. Hubei, Sichuan, Guangxi, Henan, and Anhui are found to be the regional innovative provinces with relatively high frequency. Hubei is registered six times, followed by Sichuan, Henan, and Guangxi (four times). From the global frontier perspective, all the global innovative provinces come from non-eastern China. In the above study, we have identified the eastern region and the non-eastern region as the technology leaders and followers, respectively. An examination of the phenomenon reveals that the global innovative provinces from the non-eastern region are also technologically leading provinces due to a decreasing technology gap and location on the global frontier. Non-innovative provinces can benchmark the regional innovative provinces and global innovative provinces to improve their unified healthcare efficiency.

**Table 3:** Group and metafrontier innovators.

**Year**	**Group innovator**		**Metafrontier innovator**
	**Eastern region**	**Non-eastern region**	
2009–2010	Shanghai Jiangsu Zhejiang Shandong	Henan	
2010–2011	Shanghai Jiangsu Zhejiang Shandong Guangdong	Anhui Jiangxi Henan Hubei	Jiangxi
2011–2012	Hebei Shanghai Zhejiang Fujian Shandong Guangdong	Hubei Guangxi Sichuan Guizhou Yunnan	Sichuan Yunnan
2012–2013	–	–	–
2013–2014	Shanghai Jiangsu Shandong	Hubei Hunan	Hubei
2014–2015		Anhui Hunan	
2015–2016	Hebei Zhejiang	Henan Hubei Guangxi Sichuan	
2016–2017	Shanghai Zhejiang Guangdong	Jiangxi Hubei Chongqing Sichuan	Jiangxi Guangdong
2017–2018	Zhejiang	Anhui Guangxi	
2018–2019	Jiangsu Zhejiang	Henan Hubei Guangxi Sichuan Ningxia	Henan Hubei

## Conclusion

Research in healthcare efficiency has increasingly focused on regional differences based on the assumption of the same production technology. Yet, less research considers the technology heterogeneity. In this study, we combine the non-radial directional distance function and the meta-frontier method to construct the static indicator (HEI) and dynamic indicator (MNMHEI). Through the temporal analysis of those indicators and the decomposition of MNMHEI, we evaluate the evolution of the unified healthcare efficiency at the regional level from 2009 to 2019 and identify the sources of unified efficiency changes. The main conclusions are as follows:

The findings of the HEI support the general argument that the unified healthcare efficiency in China's economically developed eastern region is significantly higher than that in the relatively underdeveloped non-eastern region. From the perspective of temporal analysis, 2012 is considered as a watershed in the development of unified healthcare efficiency. Due to the successful coverage of universal health insurance in 2011, the unified healthcare efficiency in both eastern and non-eastern China shows notable growth from 2011 to 2012 and reaches the maximum in 2012. In addition, the two regions share similar time trends of HEI and MNMHEI during the whole study period.

The result of the average MNMHEI indicates a 2.68% annual increase in unified healthcare efficiency from 2009 to 2019. Both eastern and non-eastern regions show an upward trend, with an average growth rate of 3.53 and 2.22%, respectively. The decomposition of MNMHEI reveals that the increase in unified healthcare efficiency in eastern China is mainly driven by technological progress, measured by BPC. On the contrary, the main reason for the increase in unified healthcare efficiency in non-eastern China is the better catch-up performance, measured by EC. According to TGC, the eastern region maintains the leader of technology, and the non-eastern region, as a follower, shows a narrowing technology gap.

The temporal analysis of dynamic indicator shows that the BPC time trends approximately coincide with the MNMHEI time trends in two regions, implying that technological progress has made a greater contribution to the changes in MNMHEI. Time trends of EC values in two regions show fluctuations, without obvious upward or downward trends. BPC values show similar temporal trends in the eastern and non-eastern regions and reach the maximum during 2011–2012. With the universal health insurance coverage in 2011, the increase in medical services may promote technological progress. Compared with the EC and TGC, the impact of policies on technological progress is more significant. The BPC values of the eastern region are higher than those of the non-eastern region from 2009 to 2011, whereas this gap has disappeared gradually after 2012. Interestingly, although the non-eastern region has a lower average value of BPC, it shows better growth momentum from the perspective of time trends. In general, the reform has a greater impact on the technological progress in the non-eastern region than in the eastern region.

On the whole, eastern China and non-eastern China share similar time trends of HEI and MNMHEI under the influence of policies. The unified healthcare efficiency has increased significantly from 2009 to 2019, but there are notable differences between the two regions. The emphasis on policies adopted by the two regions should be different. The eastern region is considered as a technology leader, with higher unified healthcare efficiency, better technological progress performance, and the production technology closest to the meta-frontier technology. According to the results, the eastern region should focus more on the improvement in resource management level in the production process and plays a leading role nationwide.

It cannot be ignored that the production technology in non-eastern China lags behind that in eastern China. However, the impact of the reform on the non-eastern region is more significant, as shown in the following. As a technology follower, the non-eastern region shows a decreasing technology gap and a stronger growth momentum of technological progress during the study period. We also have an interesting finding that all the global innovative provinces come from non-eastern China. From the perspective of time trends, EC shows uncertainty whereas BPC experiences a stable growth momentum. The non-eastern region should commit to improving innovation and introducing advanced technologies that are common in the eastern region. In addition, more attention should be paid to the radiation and the leading role of innovative provinces.

This study has some limitations that can be further researched. First, the group classification is based on the previous research that the eastern region shows better performance in healthcare efficiency. Future research can consider a wider range of group classifications to explore the regional heterogeneity. Second, the bootstrap methods can be incorporated to perform the statistical inference for the unified healthcare efficiency and its dynamic changes as well as the decompositions.

## Data Availability Statement

Publicly available datasets were analyzed in this study. This data can be found at: https://data.cnki.net/yearbook/Single/N2022010155.

## Author Contributions

BG contributed to conceptualization and writing—original draft. XF contributed to data curation and policy suggestions. JZ contributed to writing, reviewing, and editing the manuscript. All authors contributed to the article and approved the final manuscript.

## Funding

This work was funded by National Natural Science Foundation of China (72071121) and Shandong University Multidisciplinary Research and Innovation Team of Young Scholars (2020QNQT017).

## Conflict of Interest

The authors declare that the research was conducted in the absence of any commercial or financial relationships that could be construed as a potential conflict of interest.

## Publisher's Note

All claims expressed in this article are solely those of the authors and do not necessarily represent those of their affiliated organizations, or those of the publisher, the editors and the reviewers. Any product that may be evaluated in this article, or claim that may be made by its manufacturer, is not guaranteed or endorsed by the publisher.
